# Gut bacteria facilitate pollinivory of the ladybird beetle *Micraspis discolor*

**DOI:** 10.3389/fmicb.2024.1475985

**Published:** 2024-11-19

**Authors:** Guannan Li, Yu-Hao Huang, Li-Qun Cai, Qian Mou, Yuan-Sen Liang, Yi-Fei Sun, Hao Li, Kun-Yu Yang, Hao-Sen Li, Hong Pang

**Affiliations:** State Key Laboratory of Biocontrol, School of Ecology, Sun Yat-sen University, Shenzhen, China

**Keywords:** pollen, bacteria, antibiotics, biological control, ladybird beetle

## Abstract

The ladybird beetle *Micraspis discolor* plays an important role as a predator of various arthropods within Asia’s rice ecosystems. While pollen could serve as an alternative diet for this beetle, facilitating mass-rearing, its pollinivory might inadvertently result in attacks on crop pollen. This study aims to explore the role of gut bacteria on pollinivory of *M. discolor*. We found that antibiotic treatment significantly reduced the performance of *M. discolor* when fed *Brassica campestris* pollen. However, the treatment did not significantly affect their performance when fed an alternative diet of *Ephestia kuehniella* eggs. Further, we found that antibiotics can eliminate a strain of *Serratia marcescens*, SmMd, which is a specific gut bacterium in *M. discolor*. Moreover, the performance of *M. discolor* showed some degree of recovery when SmMd was reintroduced into its diet. Therefore, we propose that gut bacteria, particularly SmMd, play a significant role in pollen use by this ladybird beetle. This insight enhances our understanding of the important role of gut bacteria in insect adaptation to diverse diets and can potentially optimize the utilization of *M. discolor* in biological control strategies.

## 1 Introduction

Ladybird beetles are renowned for their beneficial role in pest biological control. Indeed, various species of ladybirds exhibit diverse feeding behaviors, including predation on arthropods, as well as mycophagy and herbivory (Giorgi et al., 2009). However, the intricate feeding habits of ladybirds present two key challenges in their biological control application. Firstly, augmentative biological control, which involves releasing large quantities of natural enemies into fields, necessitates a costly three-tiered artificial rearing system comprising predators, prey, and the prey’s host plants (De Clercq et al., 2011). Secondly, some ladybirds are generalists, consuming not only target pests but also other arthropods or even plant tissues, posing environmental risks (Louda et al., 2003). The most notable example is the Harlequin ladybird, *Harmonia axyridis*, employed for aphid control but also recognized as a major invasive species in introduced regions, partly due to its non-target impacts (Koch, 2003). Therefore, achieving cost-effective and environmentally safe utilization of ladybirds in biological control demands a deeper understanding of the mechanisms underpinning their feeding habits.

*Micraspis discolor*, a prevalent ladybird species found in rice fields across Asia, exhibits a broad diet, preying on various rice pests such as aphids, plant hoppers, thrips, and leafhoppers (Rattanapun, 2012). Additionally, *M. discolor* has been observed consuming pollens of rice and *Brassica campestris* (Rattanapun, 2012; Du et al., 2021). Unlike other predatory insects that typically resort to pollen consumption only during food shortages, *M. discolor* has demonstrated the capability to sustain development and reproduction solely through pollen consumption under controlled laboratory conditions (Rattanapun, 2012; Du et al., 2021), highlighting its pollinivory ability. While pollen consumption offers a rich nutrient source, facilitating population maintenance, it also poses potential risks to crops like rice. Indeed, *M. discolor* was considered a pest in rice ecosystems in China since it heavily fed on rice pollen (Wang, 1959; Gao, 1965). On the other hand, the pollinivory of *M. discolor* may allow to sustain its populations in times of prey scarcity. Thus, a comprehensive understanding of its pollen feeding habits should help to optimize its use in biological control.

Pollen serves as a vital nutrient source for various insects, containing essential amino acids, fructose, glucose, and lipids conducive to growth (Lundgren and Wiedenmann, 2004; Roulston and Cane, 2000). However, pollen employs a range of physiological and chemical defenses to deter pollinivorous insects, thereby challenging their digestion and detoxification capabilities. The intine, or inner walls of pollen, represents a significant barrier to nutrient absorption, containing indigestible plant polymers such as cellulose, pectin, and hemicellulose (Roulston and Cane, 2000; Lundgren, 2009). In pollen-consuming bees, endogenous cellulases within the glycoside hydrolase family 9 (GH9) facilitate cellulose digestion in the intine (Kunieda et al., 2006). In addition to endogenous cellulase genes, gut bacteria in bees, such as *Bifidobacterium* and *Gilliamella*, have also been found to help bees degrade polysaccharides like pectin and hemicellulose in pollen walls (Zheng et al., 2019). Nonetheless, the feeding and digestion mechanisms of pollinivorous predators like *M. discolor* remain elusive.

In our previous study, we aimed to determine whether the ability of *M. discolor* to digest pollen originated from endogenous genes or symbionts. To achieve this, we conducted a comparative analysis of the transcriptome and gut microbiota of *M. discolor* when fed on the *Ephestia kuehniella* eggs, *B. campestris* pollen, aphids, and mealybugs (Huang et al., 2022). The transcriptome comparison across different diet treatments of *M. discolor* revealed that differentially expressed genes were primarily related to nutrient metabolism, highlighting differences in nutrient and antinutrient composition among the various diets (Huang et al., 2022). In the gut microbiota comparison, several candidate cellulolytic bacteria were identified as discriminative biomarkers of pollen feeding (Huang et al., 2022). We thus hypothesized that the pollen-feeding ability of *M. discolor* is facilitated not only by endogenous factors, such as enzymes that secreted by insects, but also by its gut microbiota.

To test this hypothesis, we employed *M. discolor* and *B. campestris* pollen as a model in the present study. We first confirmed that *M. discolor* exhibits superior adaptation to a pollen diet compared to several related predatory ladybird species. Oral ingestion of the antibiotic gentamycin significantly reduced the performance of *M. discolor* when fed pollen, while this treatment did not significantly affect their performance when fed an alternative diet of *E. kuehniella* eggs. Furthermore, the antibiotic treatment consistently eliminated a strain of *Serratia marcescens*, SmMd, which is a specific gut bacterium in *M. discolor*. Reintroducing SmMd to *M. discolor* partially restored its fitness. These findings indicate a facilitative role of gut microbiota, such as SmMd, in *M. discolor*’s pollinivory.

## 2 Materials and methods

### 2.1 Performance of four ladybird species in pollen feeding

Four Coccinellini ladybird species, including *M. discolor*, *Propylea japonica*, *Cheilomenes sexmaculata*, and *Coccinella septempunctata*, were used to test larval survival rate when fed pollen. *M. discolor* was collected from Hainan Province, China, in 2018 and subsequently reared on a composite diet of *B. campestris* pollen and *E. kuehniella* eggs. *P. japonica* and *C. sexmaculata* were collected from Guangdong Province, China, in 2018, and *C. septempunctata* was collected from Henan Province, China, in 2019. *P. japonica*, *C. sexmaculata* and *C. septempunctata* were reared on the aphid *Megoura crassicauda* or *Aphis craccivora* as their diet. At the beginning of the experiment, ladybird eggs were collected, and newly emerged first-instar larvae were placed in Petri dishes (35 mm diameter, 10 mm height) with an ample supply of *B. campestris* pollen and water. The number of individuals that developed into adults was recorded, with 90–99 individuals tested for each species. All experiments were conducted in climate-controlled chambers at 25 (±1) °C, 75 (±5) % RH, under a 16:8 (L:D) photoperiod.

### 2.2 Performance of *Micraspis discolor* after antibiotic treatment

We first screened effective antibiotics for eliminating bacteria in the gut of *M. discolor* based on the method in Visôtto et al. (2009). Detailed methods for antibiotic screening were described in Supplementary material. Following antibiotic screening, gentamicin, which exhibited the largest inhibition zone among the four tested antibiotics (ampicillin: 4 mm, tetracycline: 7 mm, penicillin: 10 mm, and gentamicin: 15 mm), was selected for subsequent experiments.

To explore the effect of antibiotics on the performance of *M. discolor*, life history traits under four treatments (Treatments A, B, C, and D, Table 1) were surveyed. Each treatment comprised 61–92 individuals. Treatments A and B involved ladybirds solely fed *E. kuehniella* eggs and *B. campestris* pollen from the first instar, respectively. Individuals in Treatments C and D were initially fed *E. kuehniella* eggs or *B. campestris* pollen, then were fed antibiotics-mixed diets during the second-instar larval stage for 4 days, and subsequently returned to a diet of antibiotics-free *E. kuehniella* eggs or *B. campestris* pollen. Antibiotics-mixed diets were prepared by incorporating 50 μL of a 10 μg/mg gentamicin solution into the *E. kuehniella* eggs. This method was chosen because a preliminary experiment showed that *M. discolor* did not consume pollen mixed with antibiotics. Diets were replenished daily. Larval development time, survival rate, and weight of newly emerged adults were recorded.

**Table 1:** Treatments set of *Micraspis discolor* in this study.

Treatment ID	No. tested individual	Main diet	Antibiotic treatment	SmMd reintroduction	Experiments
A	91	*E. kuehniella* eggs	No	No	Life history
B	91	*B. campestris* pollen	No	No	Life history, feces, 16S
C	82	*E. kuehniella* eggs	Yes	No	Life history
D	81	*B. campestris* pollen	Yes	No	Life history, feces, 16S
E	66	*B. campestris* pollen	Yes	Yes	Life history, feces
F	61	*B. campestris* pollen	Yes	No, LB control	Life history

Antibiotic treatment was performed by fed antibiotics-mixed *Ephestia kuehniella* eggs during the second-instar larval stage for 4 days. SmMd reintroduction was performed by fed SmMd-mixed pollen in the third-instar larval stage for 2 days, and LB-control was performed by fed LB-mixed pollen.

The weight and morphology of *M. discolor* feces under antibiotic treatment were also examined. Third-instar larvae from Treatments B and D were randomly selected for the following tests. Ten groups were established for each treatment, each consisting of 20 individuals and 50 mg of *B. campestris* pollen, placed in Petri dishes (100 mm diameter, 10 mm height). Before the test, individuals were starved for 24 h day. After all the pollen was consumed and defecation ceased (~3 days), the feces were weighed and observed using scanning electron microscopy (SEM).

Statistical analyses for comparing life history traits and fecal weight among different treatments were carried out using R software v4.2.3. The Shapiro–Wilk normality test and Bartlett’s test were employed to assess the normality and homogeneity of variance for the developmental time and adult weight data. Data conforming to these assumptions underwent one-way ANOVA analysis. In cases where a significant difference was observed, Tukey’s honestly significant difference (HSD) test was utilized for *post hoc* comparisons. Alternatively, the Wilcoxon signed-rank test was applied. A significance level of *p* < 0.05 was adopted for all statistical tests.

### 2.3 Microbiota of *Micraspis discolor* after antibiotic treatment

To test the effect of antibiotic treatment on microbiota of *M. discolor*, fourth-instar larvae from Treatments B and D were randomly selected for 16S rRNA amplicon sequencing. Four or five samples were established for each treatment, with guts from seven individuals were pooled per one sample. Total genomic DNA was extracted using the TIANamp Genomic DNA Kit (Tiangen Biotech, Beijing, China) following the manufacturer’s instructions. A ~ 420 bp V3–V4 region of the 16S rRNA gene was amplified using primers 338F (ACTCCTACGGGAGGCAGCA) and 806R (GGACTACHVGGGTWTCTAAT). Purified PCR products were sequenced on the Illumina HiSeq 2500 platform, generating 250 bp paired-end reads. High-quality sequence reads were analyzed using QIIME v2020.06 (Bolyen et al., 2019). Reads with ≥97% similarity were clustered into operational taxonomic units (OTUs). The OTUs were then identified using the RDP classifier and reference data sets from the SILVA database (Quast et al., 2013).

Based on 16S rRNA amplicon sequencing, we found that a *S. marcescens* strain SmMd (Du et al., 2022) was eliminated by the antibiotic treatment. To confirm the effect of antibiotic treatment on SmMd elimination, SmMd-specific primers were designed for diagnostic PCR. First, the whole genome of SmMd was sequenced. Second, based on the genome of SmMd, SmMd-specific primers were designed. Third, a pair of primers, SmMd-5-87F (TTATTGCACGAAGTCAAGGG) and SmMd-5-87R (TGGGGAGAACCTACCATTTT), were selected based on PCR efficiency. This pair of primers was used for further diagnostic PCR for SmMd. Diagnostic PCR experiments were performed in the individuals from Treatments B and D, and also on the populations of other ladybird species reared in the same laboratory. Additionally, we evaluated the putative cellulolytic function of SmMd using a carboxymethyl cellulose (CMC)-based method (Kasana et al., 2008). Detail methods of the SmMd isolation, genome sequencing, PCR primer design and diagnostic PCR are described in the Supplementary material.

Carboxymethyl cellulose (CMC) sodium, commonly used as a cellulose substitute, was employed to assess the cellulolytic activity of SmMd. We utilized CMC media to evaluate the potential cellulolytic function of SmMd. CMC media contained 5.0 g/L croscarmellose sodium, 0.03 g/L yeast extract, 0.1 g/L malt extract, and 2.0 g/L CaCO_3_ (pH 7.0–7.5), following Hu et al. (2014). Agar (12.0 g/L) was added to prepare solid CMC media plates. Single bacterial colonies were cultured on CMC plates at 30°C for 4 days, with more than 10 colonies used as replicates. The plates were stained with iodine solution to observe transparent zones indicating cellulolytic activity. *Acinetobacter* sp., another bacterium isolated from *M. discolor*, was used as a negative control for comparison.

### 2.4 Performance of *Micraspis discolor* after SmMd-reintroduction

To explore the effect of SmMd reintroduction on the performance of *M. discolor*, life history traits of two treatments (Treatments E and F, Table 1) were surveyed. Individuals in Treatments E and F were initially fed *B. campestris* pollen, then were fed antibiotics-mixed diets during the second-instar larval stage for 4 days, and subsequently fed SmMd-mixed pollen or LB-mixed pollen in the third-instar larval stage for 2 days. To prepare the SmMd-mixed pollen diets, a single colony of SmMd from an LB plate was transferred and incubated in LB liquid medium at 180 rpm and 28°C for 24 h. Then, 5 μL of the bacterial solution, with a concentration of 1.3 × 10^7^ CFU/mL, was mixed with *B. campestris* pollen to create the diet for each larva. Diets were replenished daily. To confirm the colonization of SmMd after reintroduction, diagnostic PCR for SmMd was performed on individuals from Treatment E 2 days after reintroduction. Life history traits, fecal weight and morphology, and statistical analyses were performed as previously described.

## 3 Results

### 3.1 Performance of pollen-fed *Micraspis discolor* was affected by antibiotic treatment

Among the four predatory ladybird species fed *B. campestris* pollen, only *M. discolor* exhibited a high survival rate (84.58% of individuals developed into adults) and reproductive capability (Supplementary Table S1). The survival rates for the other three species, *P. japonica*, *C. sexmaculata*, and *C. septempunctata*, were relatively low (18.16, 16.31, and 0%, respectively), and none of them reproduced (Supplementary Table S1).

**Figure 1:**
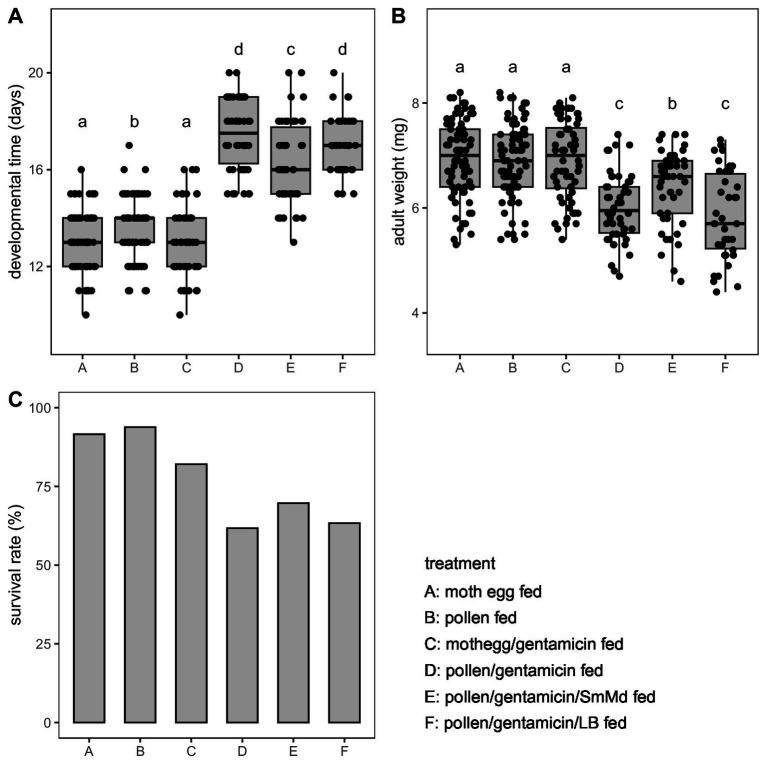
Comparison of life history traits including (A) developmental time, (B) adult weight, and (C) survival rate of *Micraspis discolor* in different treatments. Boxes with the same letter are not significantly different (*p* ≥ 0.05).

Comparing the performance of *M. discolor* across four diet treatments (Treatments A, B, C, and D) (Figure 1), individuals fed *E. kuehniella* eggs (Treatments A and C) exhibited the shortest development time, highest adult weight, and a high survival rate (>80%). The *B. campestris* pollen-fed and antibiotics-free treatment (Treatment B) had a significantly longer development time (*p* = 0.009 and 0.006 in Wilcoxon signed-rank test) but similar adult weight (*p* = 0.740 and 0.938 in Wilcoxon signed-rank test) and survival rate compared to those fed *E. kuehniella* eggs (Treatments A and C). Antibiotic treatment significantly prolonged development time and reduced adult weight when fed *B. campestris* pollen (Treatment B vs. D, development time: *p* < 0.001 and adult weight: *p* < 0.001 in Wilcoxon signed-rank test). Survival of Treatment D (62%) was also much lower than those of Treatments A, B and C (>80%). In contrast, no significant difference of traits was observed between individuals fed antibiotics-free and antibiotics-free *E. kuehniella* eggs (Treatment A vs. C, development time: *p* = 0.566 and adult weight: *p* = 0.837 in Wilcoxon signed-rank test).

**Figure 2:**
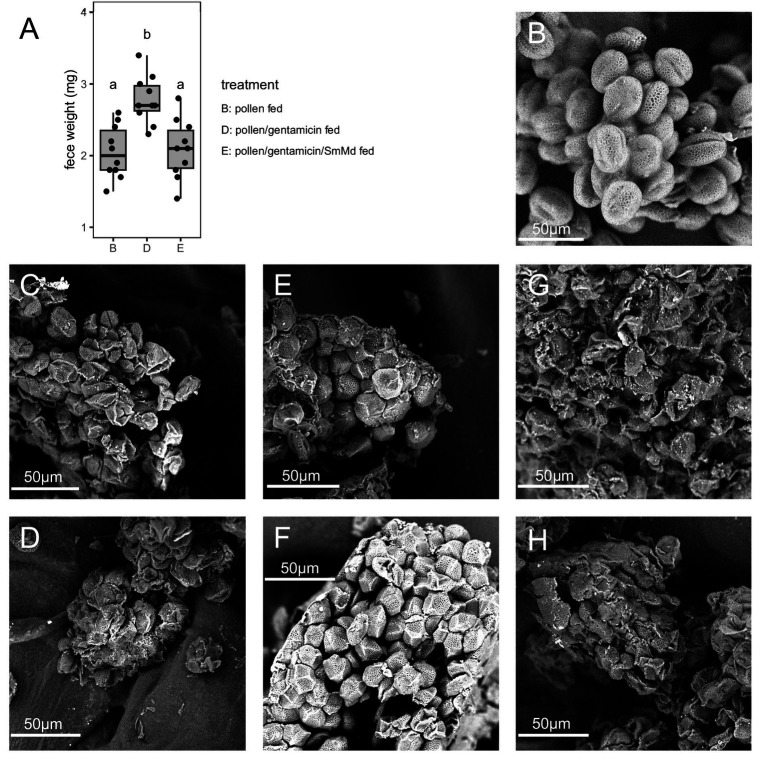
Comparison of fecal weight and morphology of *Micraspis discolor* in different treatments. (A) Comparison of fecal weight. Boxes with the same letter are not significantly different (*p* ≥ 0.05). SEM observations of the morphologies of *Brassica campestris* pollen (B) before ingestion, and in the feces of (C,D) Treatment B, (E,F) Treatment D, and (G,H) Treatment E.

In the comparison of fecal weight and morphology, the fecal weight of Treatment B was significantly lower than that of Treatment D (*p* = 0.001 in Wilcoxon signed-rank test) (Figure 2A). SEM observations showed that *B. campestris* pollen before ingestion was flattened and elliptical, with clear textures on the pollen walls (Figure 2B). The pollen in the feces of Treatment B were severely damaged and irregularly shaped (Figures 2C,D). In contrast, many of the pollen grains in the feces of Treatment D were relatively intact and maintained a similar elliptical shape to the pollen before ingestion (Figures 2E,F).

### 3.2 Antibiotic treatment eliminated SmMd in the gut of *Micraspis discolor*

**Figure 3:**
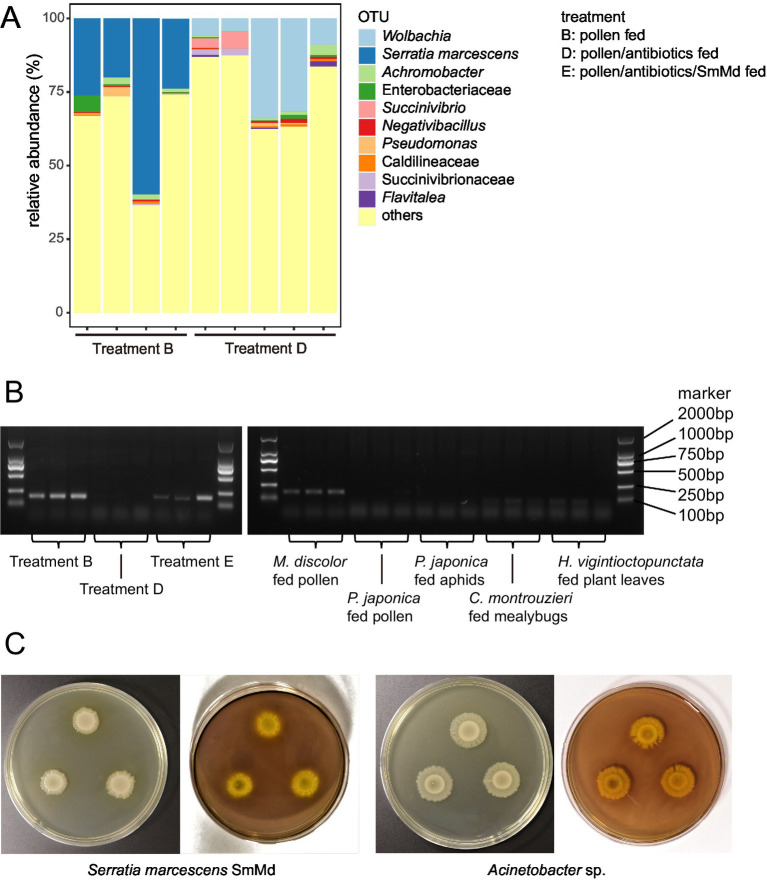
Tests of distribution and CMCase function of *Serratia marcescens* strain SmMd. (A) Relative abundance of operational taxonomic units (OTUs) in the 16S rRNA amplicon sequencing in different treatments of *Micraspis discolor*. (B) Diagnostic PCR of SmMd in different treatments of *M. discolor* and the other ladybird species reared in the same laboratory. (C) SmMd and *Acinetobacter* sp. colonies grown in CMC medium. Left: before and right: after iodine stained. The colonies were stained with iodine solution to observe transparent zones, which indicate cellulolytic activity.

Comparison of 16S rRNA amplicon sequencing data (Figure 3A) revealed that the most abundant operational taxonomic unit (OTU) in Treatment B was annotated as *S. marcescens*. This OTU had a relative abundance ranging from 20–70% in the samples of Treatment B but was not detected in the samples of the Treatment D. The most abundant OTU in Treatment D was annotated as *Wolbachia*, which was not detected in Treatment B. Most other OTUs, such as *Achromobacter* sp., *Succinivibrio* sp. and *Pseudomonas* sp., were present in samples of both treatments.

The sequenced genome of SmMd isolated from the gut of *M. discolor* had a completeness of 99.86%, no contamination, and no strain heterogeneity, indicating high quality. The 16S rRNA sequence from this genome was 100% identical to the OTU *S. marcescens* found in the 16S rRNA amplicon sequencing data, suggesting that the OTU *S. marcescens* belongs to the SmMd strain. Phylogenetic analysis showed that SmMd was closely related to human hospital pathogens (Supplementary Figures S1, S2). SmMd-specific diagnostic PCRs showed that all samples from Treatment B were positive, while all samples from Treatment D were negative (Figure 3B). Diagnostic PCRs also indicated that samples from ladybird species other than *M. discolor*, reared under the same laboratory conditions, were negative (Figure 3B). Additionally, SmMd colonies exhibited a weak degradation zone in the CMCase test (Figure 3C), and one candidate cellulase gene, GH8 (endoglucanase EC: 3.2.1.4), was identified in the SmMd genome, indicating its CMCase function.

### 3.3 SmMd-reintroduce recover the performance of antibiotic treatment

In the SmMd diagnostic PCR of the SmMd reintroduction treatment (Treatment E), all samples tested positive, indicating successful recolonization of SmMd after antibiotic elimination (Figure 3B). Comparing performance between the SmMd reintroduction treatment and the control (Treatment F) (Figure 1), SmMd reintroduction significantly increased adult weight (*p* = 0.002 in Wilcoxon signed-rank test) and shortened development time (*p* = 0.026 in Wilcoxon signed-rank test). No significant differences in development time and adult weight were detected between Treatment D and Treatment F. However, the development time and adult weight of both Treatments E and F were significantly longer and lighter, respectively, compared to Treatment B. Survival rates of Treatments D, E and F were close (60–70%) and all lower than that of Treatment B.

In the comparison of fecal weight and morphology, the fecal weight of Treatment E was significantly lower than that of Treatment D (*p* = 0.002 in Wilcoxon signed-rank test) and similar to Treatment B (*p* = 0.879 in Wilcoxon signed-rank test) (Figure 2A). The pollen in the feces of Treatment E were severely damaged and irregularly shaped (Figures 2G,H), similar to those in Treatment B.

## 4 Discussion

### 4.1 Gut bacteria facilitate pollinivory of *Micraspis discolor*

We found that *B. campestris* pollen is a suitable diet for *M. discolor*, but not for the other predatory ladybird species tested. *M. discolor* fed *B. campestris* pollen exhibited a high survival rate (>80%) and was capable of reproduction. In contrast, the other species, including *P. japonica*, *C. sexmaculata*, and *C. septempunctata*, had very low survival rates (<20%) and did not reproduce. All four ladybird species belong to the tribe Coccinellini and primarily feed on aphids. Notably, *M. discolor*, *C. sexmaculata*, and *C. septempunctata* belong to the same clade (Tomaszewska et al., 2021). Some other Coccinellini species also fed on pollen but exhibit varied performances. For example, *Coleomegilla maculata* and *H. axyridis* fed pollen were able to develop and reproduce (Lundgren and Wiedenmann, 2004; Berkvens et al., 2008), while pollen was not supported development and reproduction of *Adalia bipunctata* (He and Sigsgaard, 2019). This suggests that the ability of *M. discolor* to feed on pollen might be a recently derived trait, possibly driven by rapid evolution in ladybird genes and/or hanges in their microbiota.

We previously have found that diversity of microbiota of *M. discolor* was affected by diet used, suggesting a potential role of microbiota in shaping feeding habits (Huang et al., 2022). In this study, we investigated the role of microbiota in the pollinivory of *M. discolor* using an antibiotic-based method. Antibiotic treatment significantly impaired the performance of *M. discolor* on a pollen diet, as evidenced by prolonged development time, reduced survival rate, and decreased adult weight (Treatment D vs. Treatment B). Notably, during the antibiotic treatment period, larvae in Treatment D were fed *E. kuehniella* eggs mixed with antibiotics for 4 days, as they did not consume antibiotics-mixed pollen. Despite this, the performance of larvae in Treatment D was still inferior to those fed antibiotic-free pollen (Treatment B). Thus, we consider the influence of short-term *E. kuehniella* egg feeding during antibiotic treatment to be minimal. Additionally, antibiotic treatment increased fecal weight and the intactness of pollen in feces when fed the same amount of pollen, likely due to decreased absorption and a diminished ability to digest pollen. To assess the possible direct impact of antibiotics on the gut itself of *M. discolor*, we used individuals fed *E. kuehniella* eggs, another suitable diet for *M. discolor*, as a control. The antibiotic treatment had no significant effect on the performance of ladybirds fed *E. kuehniella* eggs (Treatment C vs. Treatment A), indicating that the direct impact of antibiotics on the gut itself was minimal. These findings suggest that certain gut bacteria, affected by antibiotic treatment, play a crucial role in facilitating the pollinivory of *M. discolor*.

### 4.2 The role of SmMd on *Micraspis discolor* in pollen feeding

Based on 16S rRNA amplicon sequencing, we found that antibiotic treatment eliminated a strain of *S. marcescens*, SmMd, from the gut of *M. discolor*. This elimination was confirmed using a SmMd-specific diagnostic PCR. The elimination of SmMd coincided with a decline in the performance of pollen-fed *M. discolor* under antibiotic treatment, leading us to hypothesize that SmMd plays a crucial role in the pollinivory of *M. discolor*. This hypothesis was partially supported by a reintroduction experiment. The performance of *M. discolor* improved significantly in the SmMd reintroduction treatment, as evidenced by decreased development time and increased adult weight (Treatment E vs. Treatment D/F). Additionally, SmMd exhibited cellulase (CMCase) activity *in vitro*, as confirmed by a CMCase test, and containing a cellulase gene in its genome, indicating its potential role in breaking down pollen walls, which may facilitate the digestion and utilization of pollen by *M. discolor*. However, the performance in the SmMd reintroduction treatment was still significantly lower than in the non-antibiotic treatment (Treatment E vs. Treatment B), suggesting that SmMd might not be the only bacterium facilitating the pollinivory of *M. discolor*.

Strains of *S. marcescens* and other *Serratia* species have been reported to exhibit various functions in their hosts (Petersen and Tisa, 2013; Shikov et al., 2023). In insect hosts, many *S. marcescens* strains act as opportunistic pathogens (Raymann et al., 2018; Zhong et al., 2023), while some provide benefits, primarily through protective functions. For instance, a strain of *S. marcescens* can confer resistance to *Plasmodium berghei* infection in mosquitoes (Bai et al., 2019), and another strain mediates detoxification of organophosphate pesticides in the bean bug *Riptortus pedestris* (Xia et al., 2023). In this study, we did not test the protective functions of SmMd as the tested *B. campestris* pollen appears to be pathogen-free (Huang et al., 2022). Instead, we report weak CMCase activity in SmMd, suggesting its cellulolytic function in degrading pollen walls. Similarly, a strain of *S. marcescens* from the gut of the bamboo-fed beetle *Cyrtotrachelus buqueti* has been shown to exhibit cellulolytic capability in degrading bamboo biomass (Tang et al., 2022). Cellulolytic activity has also been reported in a strain of *S. marcescens* isolated from avocado peel waste (Haile et al., 2022). To better understand the role of SmMd in the pollinivory of *M. discolor*, further studies are needed to elucidate the function of SmMd and its interactions with *M. discolor*.

In addition to SmMd, antibiotic treatment likely influenced other bacteria in *M. discolor*. The 16S rRNA amplicon sequencing revealed that most bacterial OTUs were present in samples from both treatments, indicating they were not sensitive to the antibiotics used. However, *Wolbachia*, the most abundant bacterium in the antibiotic treatment (Treatment D), was not detected in the antibiotics-free treatment (Treatment B). *Wolbachia* is an intracellular symbiotic bacterium that may not be easily eliminated by antibiotics. It becomes the most abundant bacterium after the antibiotics eliminate the original dominant bacteria. The role of SmMd in *M. discolor*’s pollen feeding has been demonstrated through the reintroduction treatment, while the role of *Wolbachia* in contributing to the pollinivory of *M. discolor* remains unknown.

### 4.3 Perspective for control of the pollinivory of predatory ladybirds

This study demonstrated that gut bacteria in *M. discolor* facilitate its pollinivory. The elimination and reintroduction of bacteria resulted in decreased and increased performance, respectively, of pollen-fed *M. discolor*. These findings not only enhance our understanding of important role of gut bacteria in insect adaptation to pollinivory but also provide insights for developing new strategies to optimize the use of *M. discolor* and other ladybirds in biological control. For example, eliminating key bacteria related to pollinivory in the gut of *M. discolor* via antibiotic ingestion before field release can reduce the risk of non-target impacts. Conversely, introducing or reintroducing key bacteria related to pollinivory can be beneficial for artificial rearing using pollen-based diets. Beyond *M. discolor*, many predatory ladybird species used in biological control, such as *H. axyridis* and *C. maculata*, can feed on pollen. Similar strategies might optimize their utilization in biological control programs.

## Data Availability

The raw reads of the microbiota and genome sequencing were deposited in NCBI under the project of PRJNA763096 and PRJNA754495, respectively.
