# Supplementary material for: Gut bacteria facilitate pollinivory of the ladybird beetle Micraspis discolor
Source: Front Microbiol. 2024 Nov 19;15:1475985. doi: 10.3389/fmicb.2024.1475985 (PMC11611818; doi:10.3389/fmicb.2024.1475985)
Supplement: Supplementary file 1 [file Supplementary_file_1.docx]

Supplementary Material

# Supplementary Data

Methods for screening antibiotics

Effective antibiotics for eliminating gut bacteria in *M. discolor* were screened based on the method by Visôtto et al. (Visôtto et al. 2009) with modifications. Four antibiotics, including gentamicin, penicillin, tetracycline, and ampicillin, were tested. Guts from five third or fourth-instar *M. discolor* larvae were dissected and washed in PBS solution. A drop of the PBS solution containing gut bacteria was incubated in 100 µl of LB liquid medium for 24 hours. The bacterial solution was then spread onto LB solid medium. A cotton pillar soaked with 10 μl of antibiotic solution at a concentration of 10 μg/mg was placed at the center of the LB solid medium. Inhibition zones were measured after 24 hours of incubation at 28 °C.

Methods for isolation of SmMd

The *Serratia marcescens* strain SmMd was isolated from M. discolor as described in our previous work (Du et al. 2022). Ladybird larvae and adults were washed with PBS, crushed with a sterile pestle in a 1.5 ml tube, resuspended in 100 μl PBS, and plated on LB solid medium at 28°C. After 1-3 days, small, yellow, translucent colonies were transferred to LB liquid medium and cultured at 28°C with continuous shaking. Bacteria were cryopreserved in 80% glycerol and stored at -80°C.

Methods for genome sequencing of SmMd

Whole genomic DNA of SmMd was extracted using the TIANamp Genomic DNA Kit. Paired-end libraries with a 300 bp insert size were prepared using the Nextera DNA Flex Library Prep Kit (Illumina, CA, USA) and sequenced on the Illumina NovaSeq 6000 platform. Raw reads were cleaned using SOAPnuke v2.1.4 (https://github.com/BGI-flexlab/SOAPnuke) to remove low-quality reads, adaptors, and reads with over 20% low-quality bases. Cleaned sequences were assembled *de novo* into contigs using IDBA-UD v1.1.3 (Peng et al. 2012). Genes were predicted using Prokka (Seemann 2014) and annotated using the eggNOG v5.0 (Huerta-Cepas et al. 2019) database with eggNOG-mapper v2(Cantalapiedra et al. 2021).

Methods for comparative genomics of SmMd and the other S*. marcescens* genomes

Average nucleotide identity (ANI) values between SmMd and 1934 published *S. marcescens* genomes from the NCBI database were calculated using FastANI v1.1 (Jain et al. 2018). Genomes with the top ten highest ANI values were selected for further comparison. OrthoFinder v2 (Emms and Kelly 2019) was used to identify orthologous genes among the selected genomes. A total of 2,672 single-copy orthologs were aligned using MAFFT v7.427 (Katoh and Standley 2013) with the L-INS-i algorithm and concatenated into a protein super-matrix. Phylogenetic trees were reconstructed using IQ-TREE v1.6.12 (Nguyen et al. 2015), with node support values estimated using the ultrafast bootstrap approach (UFBoot).

Methods for diagnostic PCR of SmMd

Specific genes unique to SmMd in the above comparative genomic analysis were used for primer design. Primers were designed and their specificity confirmed using Primer-BLAST (Ye et al. 2012) by default setting. SmMd-5-87F (TTATTGCACGAAGTCAAGGG) and SmMd-5-87R (TGGGGAGAACCTACCATTTT), targeting the DNA repair protein RadC gene, were chosen. PCR reactions were conducted under the following conditions: 94 °C for 5 min; 35 cycles of 94 °C for 30 s, 55 °C for 45 s, 72 °C for 1.5 min; and 72 °C for 10 min.

# Supplementary Figures and Tables

## Supplementary Figures


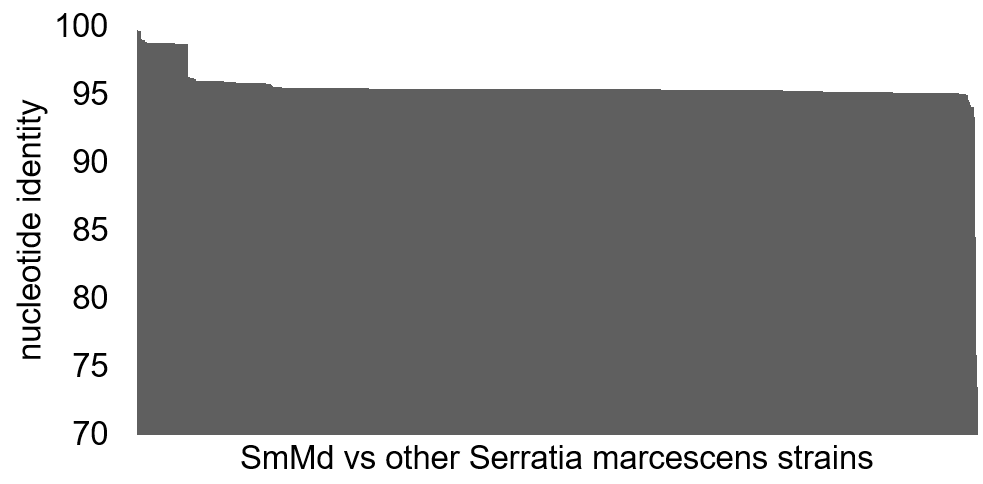


**Supplementary Figure 1.** Average nucleotide identity (ANI) of the genome of SmMd compared with the other *Serratia marcescens* genomes. Each column represents the pairwise ANI value between SmMd and other *S. marcescens* genomes, arranged from highest to lowest. Nine *S. marcescens* genomes were highly similar to SmMd, with ANI values exceeding 99%.


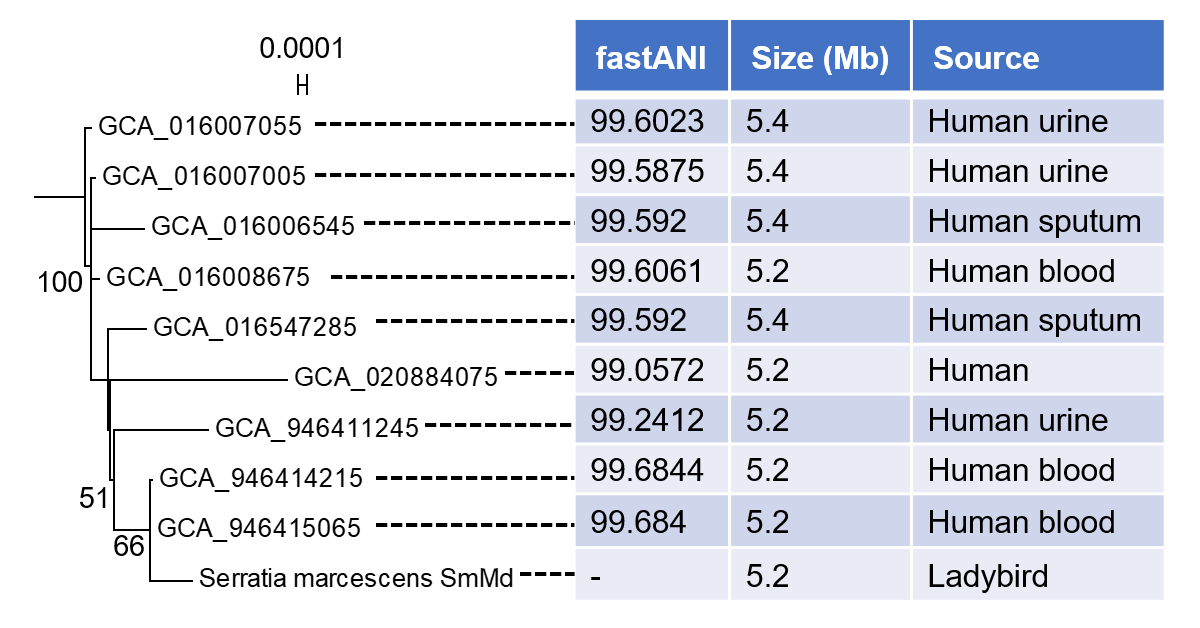


**Supplementary Figure 2.** Phylogeny and genomic comparison of the SmMd and the closely related *Serratia marcescens*. A maximum likelihood phylogenetic tree was reconstructed based on the concatenation of 2,672 single-copy orthologs shared across the tested genomes.

## Supplementary Table

**Supplementary Table 1.** Percentage of tested individuals that developed into adults. *Micraspis discolor* and three other Coccinellini species were tested.

| Species | No. tested individuals | Rate to pupa | Rate to adult | reproduction |
| --- | --- | --- | --- | --- |
| *Coccinella septempunctata* | 90 | 0% | 0% | No |
| *Cheilomenes sexmaculata* | 92 | 19.57% | 16.31% | No |
| *Propylea japonica* | 99 | 26.40% | 18.16% | No |
| *Micraspis discolor* | 91 | 84.66% | 83.58% | Yes |

# References

Cantalapiedra, C. P., A. Hernandez-Plaza, I. Letunic, P. Bork, and J. Huerta-Cepas. 2021. "eggNOG-mapper v2: Functional annotation, orthology assignments, and domain prediction at the metagenomic scale." *Mol Biol Evol* 38 (12):5825-5829. doi: 10.1093/molbev/msab293.

Du, Xue‐Yong, Huan‐Ying Yang, Sen‐Rui Gong, Pei‐Fang Zhang, Pei‐Tao Chen, Yuan‐Sen Liang, Yu‐Hao Huang, Xue‐Fei Tang, Qiao‐Kui Chen, Patrick De Clercq, Hao‐Sen Li, and Hong Pang. 2022. "Aphidophagous ladybird beetles adapt to an aphid symbiont." *Functional Ecology* 36:2593–2604. doi: 10.1111/1365-2435.14138.

Emms, D. M., and S. Kelly. 2019. "OrthoFinder: phylogenetic orthology inference for comparative genomics." *Genome Biol* 20 (1):238. doi: 10.1186/s13059-019-1832-y.

Huerta-Cepas, J., D. Szklarczyk, D. Heller, A. Hernandez-Plaza, S. K. Forslund, H. Cook, D. R. Mende, I. Letunic, T. Rattei, L. J. Jensen, C. von Mering, and P. Bork. 2019. "eggNOG 5.0: a hierarchical, functionally and phylogenetically annotated orthology resource based on 5090 organisms and 2502 viruses." *Nucleic Acids Research* 47 (D1):D309-D314. doi: 10.1093/nar/gky1085.

Jain, C., L. M. Rodriguez-R, A. M. Phillippy, K. T. Konstantinidis, and S. Aluru. 2018. "High throughput ANI analysis of 90K prokaryotic genomes reveals clear species boundaries." *Nature Communications* 9. doi: 10.1038/s41467-018-07641-9.

Katoh, K., and D. M. Standley. 2013. "MAFFT Multiple sequence alignment software version 7: Improvements in performance and usability." *Molecular Biology and Evolution* 30 (4):772-780. doi: 10.1093/molbev/mst010.

Nguyen, L. T., H. A. Schmidt, A. von Haeseler, and B. Q. Minh. 2015. "IQ-TREE: A fast and effective stochastic algorithm for estimating maximum-likelihood phylogenies." *Molecular Biology and Evolution* 32 (1):268-274. doi: 10.1093/molbev/msu300.

Peng, Y., H. C. Leung, S. M. Yiu, and F. Y. Chin. 2012. "IDBA-UD: a de novo assembler for single-cell and metagenomic sequencing data with highly uneven depth." *Bioinformatics* 28 (11):1420-8. doi: 10.1093/bioinformatics/bts174.

Seemann, T. 2014. "Prokka: rapid prokaryotic genome annotation." *Bioinformatics* 30 (14):2068-2069. doi: 10.1093/bioinformatics/btu153.

Visôtto, LE, MGA Oliveira, RNC Guedes, AOB Ribon, and PIV Good-God. 2009. "Contribution of gut bacteria to digestion and development of the velvetbean caterpillar, *Anticarsia gemmatalis*." *Journal of Insect Physiology* 55 (3):185-191. doi: 10.1016/j.jinsphys.2008.10.017.

Ye, J., G. Coulouris, I. Zaretskaya, I. Cutcutache, S. Rozen, and T. L. Madden. 2012. "Primer-BLAST: A tool to design target-specific primers for polymerase chain reaction." *Bmc Bioinformatics* 13. doi: 10.1186/1471-2105-13-134.
